# Evolving Trends in Kidney Transplant Outcomes Among Older Adults: A Comparative Analysis Before and During the COVID-19 Pandemic

**DOI:** 10.1097/TXD.0000000000001520

**Published:** 2023-11-02

**Authors:** Yiting Li, Gayathri Menon, Wenbo Wu, Amrusha Musunuru, Yusi Chen, Evelien E. Quint, Maya N. Clark-Cutaia, Laura B. Zeiser, Dorry L. Segev, Mara A. McAdams-DeMarco

**Affiliations:** 1 Department of Surgery, New York University Grossman School of Medicine, New York, NY.; 2 Department of Population Health, New York University Grossman School of Medicine, New York, NY.; 3 Department of Medicine, New York University Grossman School of Medicine, New York, NY.; 4 Division of Transplant Surgery, Department of Surgery, University Medical Center Groningen, Groningen, The Netherlands.; 5 Rory Meyers College of Nursing, New York University, New York, NY.

## Abstract

**Background.:**

Advancements in medical technology, healthcare delivery, and organ allocation resulted in improved patient/graft survival for older (age ≥65) kidney transplant (KT) recipients. However, the recent trends in these post-KT outcomes are uncertain in light of the mounting burden of cardiovascular disease, changing kidney allocation policies, heterogeneity in candidates’ risk profile, and the coronavirus disease 2019 pandemic. Thus, we examined secular trends in post-KT outcomes among older and younger KT recipients over the last 3 decades.

**Methods.:**

We identified 73 078 older and 378 800 younger adult (aged 18–64) recipients using Scientific Registry of Transplant Recipients (1990–2022). KTs were grouped into 6 prepandemic eras and 1 postpandemic-onset era. Kaplan-Meier and Cox proportional hazards models were used to examine temporal trends in post-KT mortality and death-censored graft failure.

**Results.:**

From 1990 to 2022, a 19-fold increase in the proportion of older KT recipients was observed compared to a 2-fold increase in younger adults despite a slight decline in the absolute number of older recipients in 2020. The mortality risk for older recipients between 2015 and March 14, 2020, was 39% (adjusted hazard ratio [aHR] = 0.61, 95% confidence interval [CI], 0.50-0.75) lower compared to 1990–1994, whereas that for younger adults was 47% lower (aHR = 0.53, 95% CI, 0.48-0.59). However, mortality risk during the pandemic was 25% lower (aHR = 0.75, 95% CI, 0.61-0.93) in older adults and 37% lower in younger adults (aHR = 0.63, 95% CI, 0.56-0.70) relative to 1990–1994. For both populations, the risk of graft failure declined over time and was unaffected during the pandemic relative to the preceding period.

**Conclusions.:**

The steady improvements in 5-y mortality and graft survival were disrupted during the pandemic, particularly among older adults. Specifically, mortality among older adults reflected rates seen 20 y prior.

Kidney transplantation (KT) provides long-term survival and quality-of-life benefits compared to dialysis for patients with end-stage kidney disease (ESKD)^1,2^ and is the preferred method of ESKD treatment for patients of all ages.^3,4^ Older (age ≥65) KT recipients, a significant yet highly vulnerable subpopulation of KT recipients, form an increasing percentage of KT recipients, from 7% in 1999 to 21% in 2019.^5^ This observed trend may be attributed to a combination of factors, including advancements in medical technology,^6^ improvements in healthcare delivery,^7^ and changes in organ allocation system policies over time.^8^ In particular, the implementation of kidney allocation system in 2014, which included time after dialysis initiation in a candidate’s waiting time and longevity matching,^9^ brought significant improvements in access to KT.^10,11^ However, these advancements were disrupted by the unprecedented outbreak of the Coronavirus Disease 2019 (COVID-19) pandemic.

The COVID-19 pandemic exerted an adverse impact on KT referrals and procedures, especially in the initial phase.^12^ A national survey of 111 transplant centers reported that 71.8% of living-donor KT (LDKT) programs experienced full suspension of KT, while 80.2% of deceased donor KT (DDKT) programs limited their KT operations to highly sensitized and acute ESKD patients, and those without access to dialysis.^13^ These suspensions and restrictions led to changes in post-KT management and follow-up practice.^14^ Although there are a few small studies on the prognosis of KT recipients infected with COVID-19,^15–17^ there is a paucity of comprehensive evidence on how the pandemic-induced practice changes generally affected post-KT outcomes among older recipients.

Although reported improvements in survival outcomes among KT recipients have been observed over time,^18–20^ it is crucial to acknowledge the impact of age and the risk profiles of immunocompromised patients, which may have influenced patient survival, particularly during the recent COVID-19 pandemic where a higher number of complications among immunocompromised patients were expected.^21^ Specifically, older KT candidates have a greater burden of comorbidities,^22^ especially cardiovascular disease, which is the leading cause of death in this population.^23^ The United States Renal Data System 2022 Annual Data Report noted an increasing number of older adults being listed for KT, despite the prevalence of waitlisted candidates among those undergoing dialysis declining.^24^ In light of the recent changes in kidney allocation policies,^9^ increasing heterogeneity of candidate risk profiles,^25,26^ increasing burden of comorbidities,^22^ and the COVID-19 pandemic, it is critical to comprehensively evaluate the trends in post-KT outcomes among older KT recipients and provide insight into the impact of various clinical and demographic factors on these outcomes in comparison to younger KT recipients.

To inform practice and policy in this steadily rising cohort of patients, we investigated secular trends in post-KT mortality and graft failure among older KT recipients from 1990 to 2022, including the pandemic era, and compared this trend to the younger KT recipients. We also explored differences in the trends attributable to donor type (DDKT versus LDKT). Understanding the trend of post-KT outcomes over the past 3 decades can help strengthen the current KT guidelines and policies, improve access to care for the most vulnerable individuals, and ensure effective post-KT healthcare management for older recipients.

## MATERIALS AND METHODS

### Study Design

This study used data from the Scientific Registry of Transplant Recipients (SRTR). The SRTR data system includes data on all donors, waitlisted candidates, and transplant recipients in the US, submitted by the members of the Organ Procurement and Transplantation Network. The Health Resources and Services Administration, U.S. Department of Health and Human Services provides oversight to the activities of the Organ Procurement and Transplantation Network and SRTR contractors. We leveraged SRTR data to illuminate the trends in KT outcomes in both older and younger adults between 1990 and 2022, including the pandemic era (2020–2022). This study was reviewed and determined to qualify for an ethics approval exemption under i22-00146 by the Institutional Review Board at the New York University Grossman School of Medicine because these analyses are conducted on de-identified data curated by the SRTR. All methods in this study were performed in accordance with the Declaration of Helsinki.

We included 73 078 adults aged ≥65 (hereafter referred to as older KT recipients) at the time of KT who were transplanted between January 1, 1990, and December 31, 2022. We also included a cohort of 378 800 adults aged 18–64 (hereafter referred to as younger KT recipients) for a comparison of post-KT outcomes over time. If a recipient had multiple kidney-only transplants, only the first-time KT was considered. Older and younger KT recipients who had a transplant and death/graft failure occurring on the same d, or had a transplant occurring on December 31, 2022, were excluded from the study.

### Outcomes and Patient Characteristics

The primary outcomes were post-KT mortality and death-censored graft loss. To quantify trends in these outcomes, older and younger KTs recipients were separately grouped into 7 categories of consecutive eras based on their y of KT: 1990–1994, 1995–1999, 2000–2004, 2005–2009, 2010–2014, January 1, 2015 to March 14, 2020, and March 15, 2020 to December 31, 2022. The first 6 categories were designated as pre-COVID-19 pandemic onset eras; the last category (March 15, 2020 to December 31, 2022) was designated as the post-COVID-19 pandemic onset era according to the national population incidence of COVID-19 in the United States.^27^ During the pandemic era, KT referrals and procedures were significantly disrupted,^12^ thus warranting a separate era to characterize the trends during the pandemic. Additionally, recipient factors (sex, age, race/ethnicity, body mass index [BMI], hepatitis C virus status, preemptive KT, cause of ESKD, peak panel reactive antibody, the number of y on dialysis), transplant factors (human leukocyte antigen mismatches, cold ischemia time), and donor factors (race/ethnicity, hypertension, diabetes, expanded criteria donor [ECD], and donor after circulatory death) were identified based on literature to ensure that all standard factors are adjusted for in models.^28,29^

### Statistical Analysis

KT trends in older and younger adults and their cumulative age distribution were examined. The overall and interval-specific distributions of baseline characteristics were summarized with the mean, SD, median, and interquartile range (IQR) for continuous variables, and with percentage for categorical variables. The earliest (1990–1994) and latest (March 15, 2020–2022) eras were chosen along with the 2010–2014 and 2015 to March 14, 2020 eras for cross-era comparison. P-values from Kruskal-Wallis tests for continuous variables and Fisher's exact tests for categorical variables were provided for each patient characteristic.

For each era, 1-, 3-, and 5-y survival and death-censored graft survival were estimated using the Kaplan-Meier method. Cox proportional hazards models were used to calculate adjusted hazard ratios (aHR) of outcomes. Kaplan-Meier estimates and Cox models were stratified by donor type (living versus deceased). The proportional hazards assumption was tested by Schoenfeld residuals and complementary log-log plots. For the sensitivity analysis, we estimated the cumulative incidence of graft loss using the Fine-Gray sub-distribution hazards model,^30^ treating death as a competing risk.

All statistical analyses were conducted using SAS (v9.4) and Stata (v17; Stata Corp, College Station, TX). Statistical significance was defined as a 2-sided *P*-value <0.05.

## RESULTS

### Study Population

#### Older KT Recipients

Among 73 078 first-time older KT recipients from 1990 to 2022, the mean age at transplantation was 69.3 y (SD = 3.6); 36.7% were female, 20.0% were Black, 23.0% had hypertension, and 35.3% had diabetes as the cause of ESKD. The mean BMI was 28.1 kg/m^2^ (SD = 4.9), the median time on dialysis was 1.9 y (IQR: 0.2–4.0), and 24.8% received an LDKT (Table 1).

**TABLE 1.** Characteristics of older adult KT stratified by y of transplant before and during the COVID-19 pandemic

Characteristics	All older KT recipients	1990–1994	2010–2014	2015 to March 14, 2020	March 15, 2020–2022	*P* Value
	N = 73 078	N = 1961	N = 14 353	N = 19 793	N = 14 350	
Recipient factors						
Age, mean ± SD	69.3 ± 3.6	67.9 ± 2.8	69.4 ± 3.8	69.3 ± 3.6	69.5 ± 3.6	<0.001
Female (%)	36.7	33.7	36.6	37.3	37.7	0.0032
Black (%)	20.0	11.2	19.3	21.9	22.9	<0.001
BMI (kg/m^2^), mean ± SD	28.1 ± 4.9	25.1 ± 4.2	28.3 ± 4.9	28.2 ± 4.9	28.2 ± 4.9	<0.001
HCV positive (%)	4.5	2.7	3.5	6.0	5.3	<0.001
y on dialysis, median (IQR)	1.9 (0.2–4.0)	1.8 (0.9–3.0)	2.0 (0.2–4.1)	2.1 (0.1–4.6)	1.9 (0.0–4.3)	<0.001
Peak PRA, median (IQR)	0.0 (0.0–12.0)	3.0 (0.0–10.0)	0.0 (0.0–18.0)	0.0 (0.0–21.0)	0.0 (0.0–0.0)	<0.001
Cause of ESKD (%)						
Diabetes mellitus	35.3	14.7	35.7	39.0	40.8	<0.001
Hypertension	23.0	19.5	25.6	23.0	21.8	<0.001
Polycystic kidney disease	7.5	9.7	7.3	7.3	7.1	0.0004
Glomerulonephritis	12.9	25.3	12.2	12.0	11.1	<0.001
Other	21.3	30.7	19.2	18.7	19.2	<0.001
KT factors						
No human leukocyte antigen mismatches (%)	6.4	8.2	5.8	3.7	3.9	<0.001
Kidney pumped (%)^a^	42.1	11.3	44.5	50.5	58.3	<0.001
Cold ischemia time, median (IQR)^a^	15.0 (6.5–22.0)	21.0 (14.0–29.0)	13.0 (5.2–20.1)	14.1 (5.3–21.4)	17.2 (9.0–23.0)	<0.001
Donor factors						
Live donor KT (%)	24.8	10.8	25.8	25.7	20.3	<0.001
Donor age, mean ± SD	45.8 ± 14.9	36.4 ± 17.4	45.3 ± 15.4	46.1 ± 14.7	47.1 ± 13.8	<0.001
White race (%)	73.3	83.2	72.5	72.2	70.2	<0.001
Donation after circulatory death (%)^a^	13.8	0.3^b^	11.1	17.7	26.9	<0.001
ECD (%)^a^^,^^c^	29.7	12.8	32.5	26.8	27.2	<0.001

a For deceased donors.

b For 1994–1996 first y donation after circulatory death was reliably recorded.

c Expanded criteria donors refer to older kidney donors (≥60 y or 50–59 y) and have 2 of the following 3 features: hypertension, terminal serum creatinine >1.5 mg/dL, or death from cerebrovascular accident.

The *P* values compare the value of each recipient, donor, and transplant factor between 1990–1994, 2010–2014, 2015 to March 14, 2020, and March 15, 2020 to 2022.

COVID-19, coronavirus disease 2019; ECD, expanded criteria donor; IQR, interquartile range; KT, kidney transplantation.

#### Younger KT Recipients

Among 378 800 first-time younger KT recipients from 1990 to 2022, the mean age at transplantation was 46.9 y (SD = 11.9); 39.7% were female, 26.6% were Black, 17.6% had hypertension, and 24.5% had diabetes as the cause of ESKD. The mean BMI was 27.9 (SD: 5.7), the median time on dialysis was 1.8 y (IQR: 0.4–4.1), and 34.4% received an LDKT (**Table S1, SDC**, http://links.lww.com/TXD/A589).

### Trends of KT Performed Among Older and Younger KT Recipients

**FIGURE 1.**
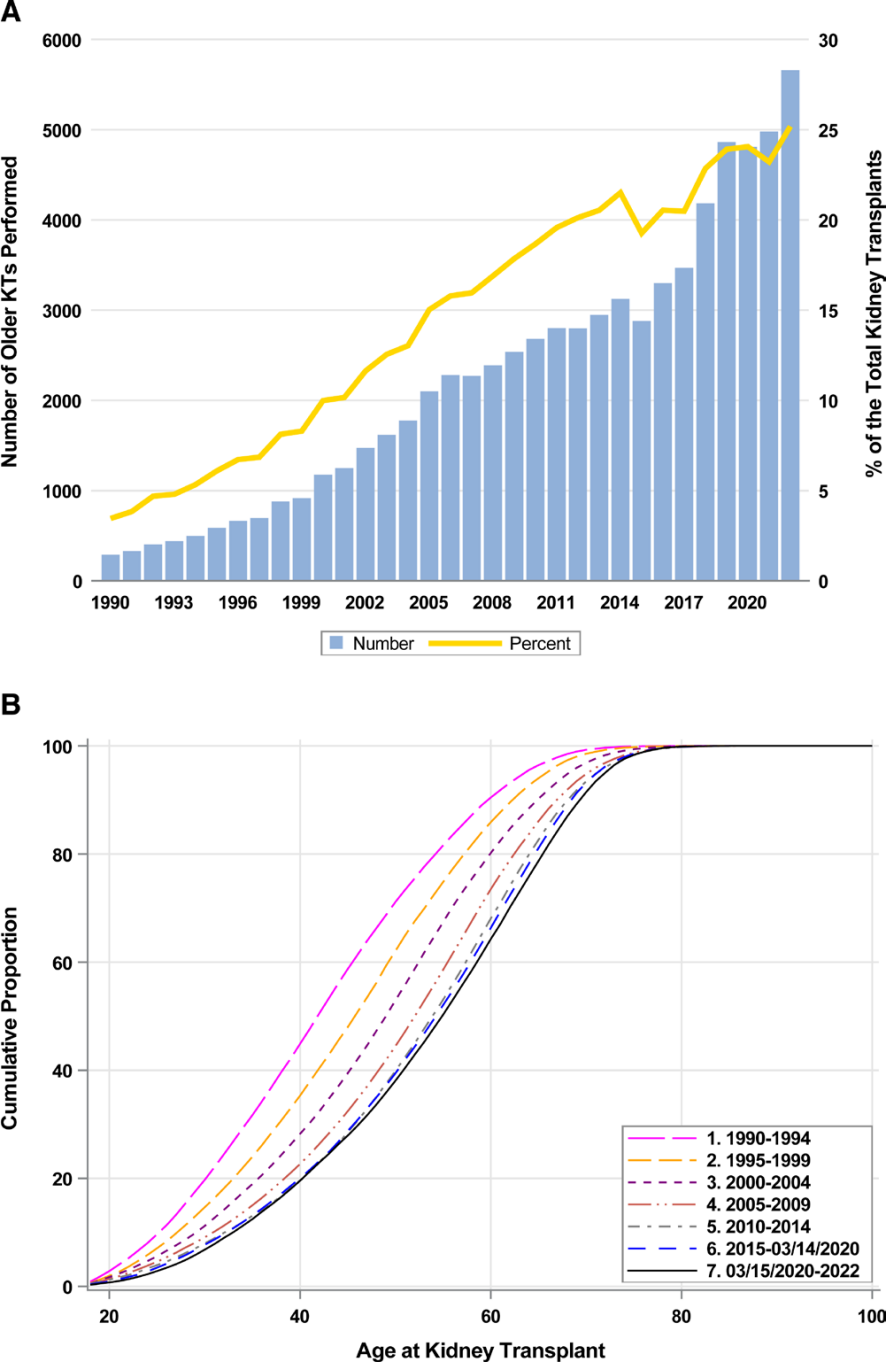
A, Older adult KT recipients stratified by the y of transplant. The left y-axis (bar) shows the number of older KT recipients, and the right y-axis (line) shows the percent of those aged ≥65 y out of the total KT recipients in that y. B, Cumulative distribution of age at first KT stratified by the y of transplant between 1990 and 2022. KT, kidney transplant.

The number of older KTs (deceased and living) increased from 290 to 3299 (20.5% of all adult KTs) between 1990 and 2016, and to 4864 (23.9% of all adult KTs) from 2017 to 2019. With the onset of the pandemic, the number of older KTs dropped to 4810 (24.0% of all adult KTs) in 2020, which increased to 5658 (25.2% of all adult KTs) in 2022 (Figure 1A). Over 5600 KTs were performed in 2022, reflecting a 19-fold increase in older KT recipients from that in 1990 (N = 290), in contrast to the 2-fold increase in KTs among younger adults during the same period (from 8097 in 1990 to 16 808 in 2022) (Figure S1). Furthermore, older adults accounted for 26% of all DDKTs in 2022, 6.5-fold higher than that in 1990 (4.0%); older adults accounted for 22.1% of all LDKTs in 2022, 14.7-fold higher than that in 1990 (1.5%). Younger adults accounted for 73.9% of all DDKTs in 2022, a 1.3-fold decrease from that in 1990 (96.0%); younger adults accounted for 77.9% of all LDKTs in 2022, 1.3-fold lower than that in 1990 (98.5%). Finally, the distribution of age at KT has gradually shifted towards older age over time (Figure 1B).

### Characteristics of Older KT Recipients

The characteristics of older KT recipients have changed over time (Table 1). From 1990 to 1994, the median age at the time of KT was 67 y, which increased to 69 y in 2015–2022. Compared to older recipients in 1990–1994, older KT recipients from 2015 to March 14, 2020, (prepandemic era) were more likely to be female (37.3% versus 33.7%, *P* = 0.003), be Black (21.9% versus 11.2%, *P* < 0.001), have higher BMI (28.2 versus 25.1, *P* < 0.001), have a longer median time on dialysis before KT (2.1 versus 1.8 y, *P* < 0.001), and have diabetes (39.0% versus 14.7%, *P* < 0.001) or hypertension (23.0% versus 19.5%, *P* < 0.001) as the cause of ESKD. Furthermore, older KT recipients between 2015 and March 14, 2020 were more likely to have received LDKTs than those between 1990 and 1994 (25.7% versus 10.8%, *P* < 0.001), received KT from a donor after circulatory death (17.7% versus 0.3%, *P* < 0.001), received KT from an ECD (26.8% versus 12.8%, *P* < 0.001) and less likely to have White kidney donors (72.2% versus 83.2%, *P* < 0.001). These characteristics were relatively consistent from the most recent prepandemic era through the pandemic era (March 15, 2020 to 2022).

### Patient Survival for Older and Younger KT Recipients

**FIGURE 2.**
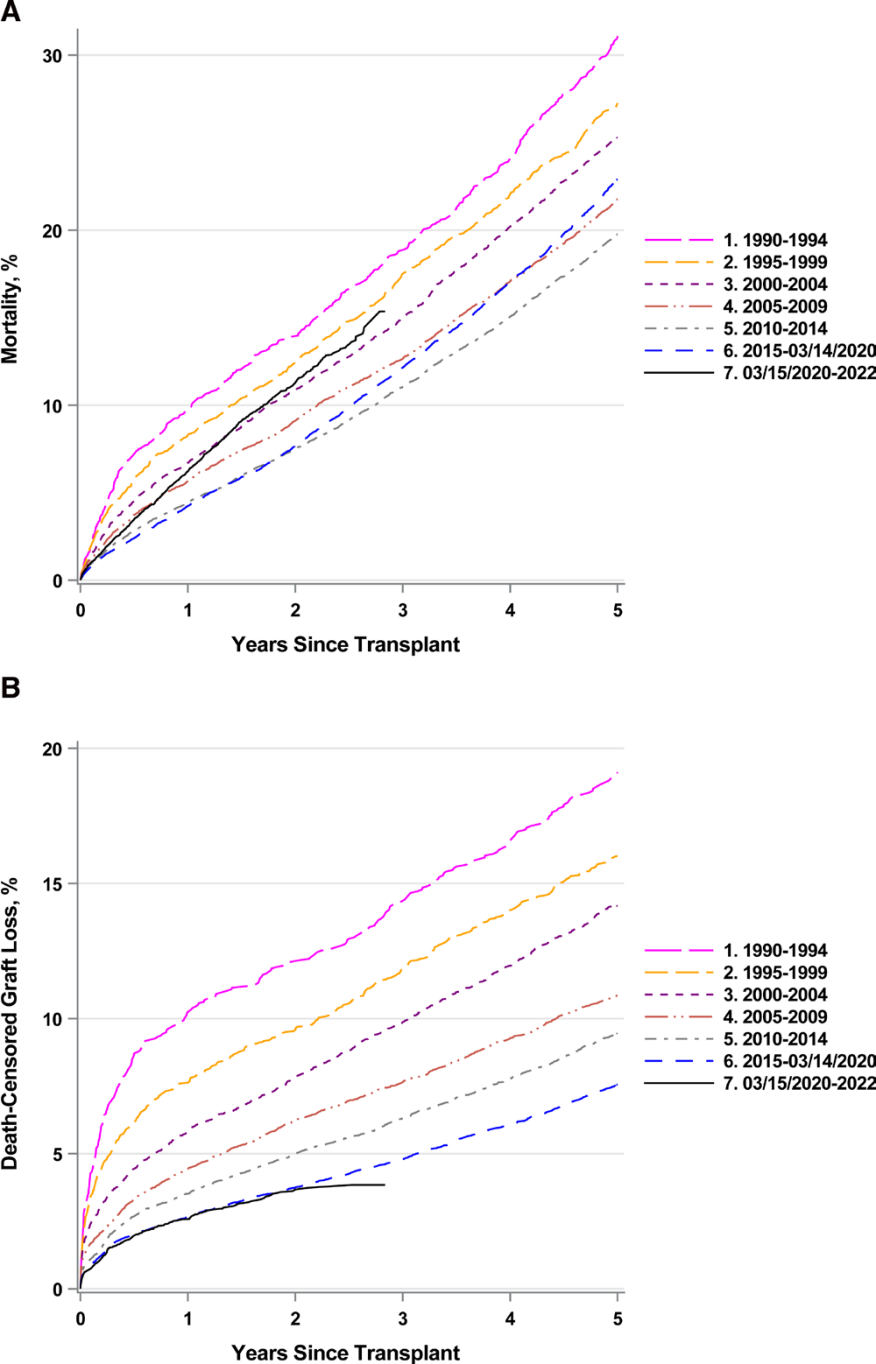
A, Mortality in older adult KT recipients, and (B) should be replaced with B, death-censored graft loss in older KT recipients by the y of transplant. Transplants during the COVID-19 pandemic era are denoted by the black colored curve, which ends near 2.75 y because of limited follow-up data in this cohort. COVID-19, coronavirus disease 2019; KT, kidney transplantation.

Before the pandemic, the 5-y survival for older KT recipients was steadily improving (Table 2). However, post-KT survival decreased after the pandemic onset in March 2020, with the overall mortality higher than that observed during the 2000–2004 period (Figure 2A Figure S2A).

**TABLE 2.** Outcomes in older KT recipients according to y of transplant

Y of KT	N	1 y	3 y	5 y	Older adults	Younger adults
		%			aHR (95% CI)	
Participant survival						
All donor (n = 73 078)^a^						
1990–1994	1961	90	81	69	Reference	Reference
1995–1999	3745	92	82	73	0.84 (0.70-1.00)	0.87 (0.80-0.93)
2000–2004	7296	93	85	75	0.76 (0.64-0.91)	0.79 (0.74-0.86)
2005–2009	11 580	94	87	78	0.61 (0.51-0.72)	0.63 (0.58-0.68)
2010–2014	14 353	96	89	80	0.53 (0.44-0.63)	0.50 (0.46-0.54)
2015 to March 14, 2020	19 793	96	88	78	0.61 (0.50-0.75)	0.53 (0.48-0.59)
March 15, 2020 to 2022	14 350	94	−	−	0.75 (0.61-0.93)	0.63 (0.56-0.70)
Deceased donor (n = 54 943)^a^						
1990–1994	1749	90	80	68	Reference	Reference
1995–1999	2963	91	81	71	0.82 (0.68-0.99)	0.87 (0.80-0.95)
2000–2004	5018	92	83	72	0.74 (0.61-0.89)	0.80 (0.73-0.87)
2005–2009	8410	93	86	76	0.59 (0.49-0.71)	0.63 (0.58-0.69)
2010–2014	10 651	95	88	79	0.50 (0.42-0.61)	0.50 (0.45-0.54)
2015 to March 14, 2020	14 712	95	86	75	0.60 (0.48-0.74)	0.52 (0.46-0.58)
March 15, 2020 to 2022	11 440	93	−	−	0.73 (0.59-0.92)	0.58 (0.51-0.66)
Live donor (n = 18 135)^b^						
1990–1994	212	92	85	77	Reference	Reference
1995–1999	782	94	88	80	0.98 (0.55-1.76)	0.83 (0.71-0.98)
2000–2004	2278	96	89	81	0.93 (0.53-1.65)	0.75 (0.64-0.89)
2005–2009	3170	97	92	84	0.72 (0.41-1.28)	0.58 (0.49-0.68)
2010–2014	3702	97	92	85	0.71 (0.40-1.26)	0.48 (0.40-0.57)
2015 to March 14, 2020	5081	98	93	85	0.71 (0.39-1.30)	0.56 (0.45-0.69)
March 15, 2020 to 2022	2910	97	−	−	0.80 (0.42-1.50)	0.70 (0.54-0.91)
Death-censored graft survival						
All donor (n = 73 078)^c^						
1990–1994	1961	90	86	81	Reference	Reference
1995–1999	3745	92	88	84	0.80 (0.63-1.02)	0.84 (0.80-0.89)
2000–2004	7296	94	90	86	0.72 (0.56-0.91)	0.75 (0.71-0.79)
2005–2009	11 580	96	92	89	0.49 (0.38-0.62)	0.60 (0.57-0.64)
2010–2014	14 353	96	94	91	0.41 (0.32-0.52)	0.47 (0.44-0.50)
2015 to March 14, 2020	19 793	97	95	92	0.31 (0.23-0.41)	0.40 (0.37-0.43)
March 15, 2020 to 2022	14 350	97	−	−	0.26 (0.19-0.35)	0.31 (0.28-0.35)
Deceased donor (n = 54 943)^c^						
1990–1994	1749	89	85	80	Reference	Reference
1995–1999	2963	91	87	82	0.77 (0.60-0.99)	0.80 (0.76-0.85)
2000–2004	5018	93	88	83	0.70 (0.55-0.90)	0.71 (0.66-0.75)
2005–2009	8410	95	91	87	0.47 (0.36-0.60)	0.57 (0.53-0.61)
2010–2014	10 651	96	93	89	0.40 (0.31-0.52)	0.44 (0.41-0.47)
2015 to March 14, 2020	14 712	97	94	91	0.31 (0.23-0.42)	0.38 (0.35-0.42)
March 15, 2020 to 2022	11 440	97	−	−	0.26 (0.19-0.36)	0.30 (0.27-0.34)
Live donor (n = 18 135)^d^						
1990–1994	212	94	92	91	Reference	Reference
1995–1999	782	95	93	91	1.46 (0.53-4.02)	0.95 (0.85-1.07)
2000–2004	2278	97	95	93	1.16 (0.42-3.19)	0.86 (0.76-0.96)
2005–2009	3170	98	96	94	0.88 (0.32-2.43)	0.70 (0.62-0.79)
2010–2014	3702	98	97	96	0.63 (0.23-1.73)	0.55 (0.49-0.62)
2015 to –March 14, 2020	5081	99	98	97	0.43 (0.15-1.25)	0.43 (0.37-0.51)
March 15, 2020 to 2022	2910	99	−	−	0.26 (0.08-0.81)	0.29 (0.23-0.37)

a Recipient factors (sex, age, race, BMI, HCV status, preemptive KT, cause of ESKD, peak PRA, number of y on dialysis), transplant factors (HLA mismatches, cold ischemia time, kidney pumped, insurance type), and donor factors (race, age, hypertension, ECD, DCD, diabetes mellitus).

b Recipient factors (sex, age, race, BMI, HCV status, preemptive KT, cause of ESKD, peak PRA, number of y on dialysis), transplant factors (HLA mismatches, insurance type), donor factors (race, age).

c Recipient factors (sex, age, race, BMI, HCV status, preemptive KT, cause of ESKD, peak PRA, number of y on dialysis), transplant factors (HLA mismatches, cold ischemia time, kidney pumped, and insurance type), donor factors (race, age, hypertension, ECD, DCD, diabetes mellitus, stroke as cause of death).

d Recipient factors (sex, age, race, BMI, HCV status, cause of ESKD, peak PRA), transplant factors (HLA mismatches, cold ischemia time, insurance type), donor factors (race, age).

Older recipients (age ≥65); column 7 represents younger recipients (age <65).

Cox models were assessed to estimate adjusted hazard ratios (aHRs) of mortality and death-censored graft loss over time relative to 1990–1994 while adjusting for recipient, donor, and transplant factors as listed below.

aHR, adjusted Hazard ratio; BMI, body mass index; CI, confidence interval; DCD, donation after cardiac death; ECD, expanded criteria donor; ESKD, end-stage kidney disease; HCV, hepatitis C virus; HLA, human leukocyte antigen; KT, kidney transplantation; PRA, panel reactive antibody.

The 1-y survival for older KT recipients improved from 90% in 1990–1994 to 96% in 2015 to March 14, 2020 (Table 2). From March 15, 2020 to 2022, the 1-y survival was 94%, identical to that in 2005–2009. Similar survival trends were observed for older and younger DDKT and LDKT recipients (Table 2). However, LDKT recipients experienced better post-KT survival than DDKT recipients, independent of recipient age.

Between 1995 and March 14, 2020, the adjusted hazard of all-cause mortality for older recipients relative to the 1990–1994 era decreased steadily (in 1995–1999, aHR = 0.84, 95% confidence interval [CI], 0.70-1.00; in 2015 to March 14, 2020, aHR = 0.61, 95% CI, 0.50-0.75). However, the risk of mortality for older recipients during the pandemic increased and was comparable to the risk of mortality in 2000–2004 (Table 2). Similarly, for younger KT recipients, the adjusted hazard of all-cause mortality decreased relative to the 1990–1994 era, from 13% lower in 1995–1999 (aHR = 0.87, 95% CI, 0.80-0.93) to 47% lower in 2015 to March 14, 2020 (aHR = 0.53, 95% CI, 0.48-0.59). During the pandemic era, the adjusted hazard of all-cause mortality was comparable to the 2005–2009 era (Table 2).

For older DDKT recipients, the mortality risk from 2015 to March 14, 2020 and pandemic eras were 40% and 27% lower than that during the 1990–1994 era, respectively (Table 2). A similar trend was seen in younger DDKT recipients. Older LDKT recipients had a 29% lower mortality risk in 2015 to March 14, 2020 era compared to 1990–1994, although the difference was not statistically significant. During the pandemic, the mortality risk was not significantly different from the 2000–2004 era. Similar trends were observed for younger LDKT recipients (Table 2).

### Death-censored Graft Survival for Older and Younger KT Recipients

Death-censored graft survival consistently improved between 1990 and March 14, 2020 (Figure 2B Figure S2B). The 1-y graft survival increased from 90% in 1990–1994 to 97% during the prepandemic era (2015 to March 14, 2020), with no further changes during the pandemic era (Table 2). Similar patterns of 1-y graft survival were observed for older and younger DDKT and LDKT recipients.

After adjusting for patient characteristics, the risk of graft loss during 2015 to March 14, 2020, and pandemic eras were 69% (aHR = 0.31, 95% CI, 0.23-0.41) and 74% (aHR = 0.26, 95% CI, 0.19-0.35) lower than 1990–1994, respectively; a similar trend was observed for younger adults (Table 2).

Similarly, the risk of graft failure in older DDKT and LDKT recipients decreased over time relative to 1990–1994; for older DDKT and LDKT recipients from 2015 to March 14, 2020, was 69% and 57% lower, respectively. The risk of graft failure for younger adult DDKT and LDKT recipients was 62% and 57% lower, respectively, relative to 1990–1994. During the pandemic era, the risk of graft failure was 74% lower for older DDKT recipients, and 74% lower for older LDKT recipients, which was comparable to that seen from 2015 to March 14, 2020 (see Table S2 for differences by sex). A similar pattern was seen in younger adult DDKT and LDKT recipients (Table 2).

## DISCUSSION

Using a national cohort, we analyzed the outcomes of 73 078 first-time KT recipients aged ≥65 y and compared them with a cohort of 378 800 younger KT recipients aged 18–64, spanning over 3 decades. Among older adults, there was a significant increase in the total number of KTs (19.0-fold), DDKTs (6.5-fold), and LDKTs (14.7-fold), compared to younger adults, who experienced a smaller increase in KTs (2-fold), DDKTs (1.3-fold), and LDKTs (1.3-fold). Despite mortality risk decreasing over time in both younger and older KT recipients, older recipients experienced a smaller reduction in absolute mortality risk than their younger counterparts, regardless of the type of transplant they received. Specifically, DDKT recipients of all ages had a higher risk of mortality during the pandemic era than in the 1990–1994 era. The risk of graft failure decreased steadily for both younger and older KT recipients over time, with a comparable trend in the reduction of graft loss observed between the 2 groups.

We found that the risk of post-KT mortality among older and younger KT recipients had been undergoing steady improvements over time, consistent with the literature.^19^ Additionally, a study reporting early- and late post-KT mortality noted improvements in the 5-y all-cause mortality across the age spectrum between 1980 and 2018.^31^ Specifically, for older adults, it was previously reported that the 5-y cumulative incidence of mortality improved from 41% to 33% in DDKT recipients and 32%–19% in LDKT recipients from 1990 to 2011.^32^ It is also worth noting that the trajectories of post-KT mortality before the pandemic are similar in older and younger recipients, despite a higher burden of comorbidities in older adults.^33^

Additionally, there had been a consistent improvement in post-KT graft failure rates for both older and younger KT recipients, evidenced by the decreasing graft loss over time reported in the 2020 United States Renal Data System Annual Data Report.^19^ For older recipients, it was previously reported that in 2009–2011, the likelihood of all-cause graft failure was 65% lower among DDKT recipients and 59% lower among LDKT recipients, respectively, than that in 1990–1993.^29^ A major factor contributing to this improvement is better donor selection. With advances in medical technology, doctors are now better equipped to evaluate and match donors with recipients, resulting in higher transplantation success rates.^34^ Additionally, improvements in surgical techniques played a significant role in increasing the safety and success of KT surgeries. The development of novel and improved immunosuppression regimens and protocol biopsies is another possible factor contributing to lower rates of graft failure by preventing organ rejection.^35–38^ Lastly, the implementation of policies such as the kidnay allocation system in 2014 has contributed to improving equity in organ allocation.^39,40^ During the pandemic era, the cumulative incidence of post-KT graft failure tracked closely with that during 2015 to March 14, 2020, era for both older and younger KT recipients, indicating that the pandemic did not seem to exert a substantial impact on post-KT graft failure. Our findings highlight the sustained success of survival outcomes following KT over time, particularly among older adults, even with increasing utilization of at-risk kidneys.^32,41,42^ Furthermore, the expanded donor pool because of the use of at-risk kidneys among older adults may account for the shorter times on dialysis compared to younger adults. These findings underscore the substantial survival advantage conferred by KT, particularly for older adults, despite the increased use of kidneys from at-risk donors.^43,44^

To the best of our knowledge, this is the first study to leverage national registry data to investigate secular trends of post-KT outcomes in older recipients, through the pandemic era, whereas previous investigations were limited to examining KT recipients either before the pandemic, or those infected with severe acute respiratory syndrome coronavirus 2 (SARS-CoV-2) if the pandemic era were included in the study period.^45,46^ Older adults remained a vulnerable population during the pandemic, with a survey reporting over a third of older adults having their medical/health appointments canceled or postponed, and over 20% of older adults affected by the cancelation of assistance with activities of daily living,^47,48^ warranting additional considerations for the provision of care in this population. This trend is more concerning in older KT recipients because of COVID-19-related cardiovascular complications,^49^ limitations on access to medical care due to pandemic-related changes,^50^ and the preexisting burden of comorbid conditions, including cardiovascular illnesses.^23^ Notably, during the pandemic era, post-KT mortality was worse than what was observed in 2000–2004 for older adults, and the 2005–2009 era for younger adults.

In our study, we also noted longer cold ischemia times in both older and younger KT recipients during the pandemic era, which can cause ischemia-reperfusion injury, leading to delayed graft function, acute/chronic graft rejection, chronic graft dysfunction, and an increased risk of post-KT mortality.^51–53^ Other contributing factors to the increased mortality rate observed include renal injury caused by viral tropism,^54^ dysregulated inflammatory responses, coagulation abnormalities, and complement cascades in response to SARS-CoV-2 infection.^55^ Older KT recipients form a unique risk group because of immunosenescence, ESKD-related immune changes,^56^ immunosuppressant use, comorbidities,^57^ and cardiovascular conditions,^58^ compounded by SARS-CoV-2 infections, putting them at greater risk for post-KT mortality and graft loss.^59,60^ Moreover, we hypothesize that both older and younger recipients with a high risk of post-KT graft failure might have experienced early death before graft loss due to a possible combination of SARS-CoV-2 infection with coexisting chronic conditions.^61–63^ In the event that many potential cases of post-KT graft failure were censored by early death, the observed rate of post-KT graft failure remained relatively stable before and during the pandemic, consistent with our findings.

Furthermore, our observations revealed a significant increase in the utilization of ECD kidneys among older KT recipients compared to their younger counterparts from 1990 to 2022. This trend is substantiated by research indicating a growing acceptance of older donors by transplant programs and a higher likelihood for older adults to accept and receive ECD donor kidneys, surpassing younger KT recipients.^41^ Interestingly, a recent study determined that ECD kidneys were associated with excess mortality among younger KT recipients, while no such association was observed among older recipients.^64^ These findings highlight the evolving landscape of KT, in which older recipients are increasingly benefiting from marginal kidneys, whereas it may not be an ideal option for younger recipients, considering the potential impact on their survival.

There are 2 major strengths of this study: the linkage of several national databases to analyze trends and outcomes of older and younger KT recipients, with a focus on the pandemic era. A limitation of our study is the inability to ascertain the causes of death, including the number of COVID-19 infections and related deaths in older and younger KT recipients during the pandemic. Because of the availability of the data, we were only able to assess recipient mortality and graft survival outcomes up to 2.75 y post-KT during the pandemic era. It should be noted that the shorter follow-up period in comparison to other periods, which extended outcomes up to 5 y, might affect the comparability of outcomes. Future research should focus on an extended follow-up period to thoroughly evaluate the long-term impact of the pandemic on both patient and graft survival. Furthermore, it is important to acknowledge that our data does not include individuals who are currently on dialysis. Consequently, we were unable to directly compare the benefits of KT to remaining on dialysis, particularly among older KT candidates. This limitation precludes us from providing insights into the comparative advantages of transplantation over dialysis for older KT candidates, especially during the COVID-19 pandemic. Nevertheless, several studies have reported on mortality post-COVID-19 diagnosis in this population, with KT recipients experiencing worse mortality outcomes.^24^ Moreover, studies have also reported increased waitlist removals due to mortality during the initial phases of the COVID-19 pandemic, which may also reflect higher mortality rates among older adults awaiting KT.^65^

## CONCLUSIONS

In summary, the proportion of older KT recipients increased from 1990 to 2019. With the outbreak of the pandemic, the proportion of older KT recipients dropped in 2020 and rebounded in 2022. This trend contrasts with what was observed in younger adults between 1990 and 2022. The positive trend in post-KT mortality was affected during the pandemic era, especially for older adult recipients. The hazard of graft failure decreased over time for both older and younger KT recipients and remained unaffected during the pandemic era. It is essential to have a comprehensive understanding of the landscape of post-KT outcomes over time for older and younger KT recipients, which can aid in strengthening measures to mitigate poor health outcomes and improve long-term results in this population. Our findings hold crucial implications for providers caring for older KT recipients, particularly in fostering informed discussions regarding the potential benefits of transplantation. This is particularly relevant in light of the growing population of older individuals who have ESKD and have undergone KT. Although our study did not directly compare outcomes between individuals on dialysis and KT recipients, it underscores the need to address the benefits of transplantation in the context of older KT recipients. Future research comparing dialysis and transplantation outcomes is warranted to provide a comprehensive understanding of the treatment options available and to support shared decision-making for older KT candidates.

## ACKNOWLEDGMENTS

The data reported here have been supplied by the Hennepin Healthcare Research Institute (HHRI) as the contractor for the SRTR. The interpretation and reporting of these data are the responsibility of the author(s) and in no way should be seen as an official policy of or interpretation by the SRTR or the U.S. Government.

## Supplementary Material


