# Supplementary material for: Evolving Trends in Kidney Transplant Outcomes Among Older Adults: A Comparative Analysis Before and During the COVID-19 Pandemic
Source: Transplant Direct. 2023 Nov 2;9(12):e1520. doi: 10.1097/TXD.0000000000001520 (PMC10624464; doi:10.1097/TXD.0000000000001520)

Supplementary Material for

Evolving Trends in Kidney Transplant Outcomes among Older Adults: A Comparative Analysis before and during the COVID-19 Pandemic

Authors:

Yiting Li MPH[1], Gayathri Menon MHS[1], Wenbo Wu PhD[2,3], Amrusha Musunuru MD[1], Yusi Chen MHS[1], Evelien E. Quint MD[4], Maya N. Clark-Cutaia PhD, ACNP-BC[3,5], Laura B. Zeiser ScM[1], Dorry L. Segev MD, PhD[1,2], Mara A. McAdams-DeMarco PhD[1,2]

[1]Department of Surgery, New York University Grossman School of Medicine, New York, NY

[2]Department of Population Health, New York University Grossman School of Medicine, New York, NY

[3]Department of Medicine, New York University Grossman School of Medicine, New York, NY

[4]Division of Transplant Surgery, Department of Surgery, University Medical Center Groningen, Groningen, The Netherlands

[5]Rory Meyers College of Nursing, New York University, New York, NY

Table of Contents:

Table S1. Characteristics of Younger Adult Kidney Transplant (KT) Stratified by Year of Transplant before and during the COVID-19 Pandemic

Table S2. Outcomes in Older Kidney Transplant (KT) Recipients by Sex

Figure S1. Younger adult kidney transplant (KT) recipients stratified by

the year of transplant. The left y-axis (bar) shows the number of younger KT recipients, and the right y-axis (line) shows the percent of those aged <65 years out of the total KT recipients in that year.

Figure S2. Mortality in younger adult kidney transplant (KT) recipients,

and (B) death-censored graft loss in younger KT recipients by the year of transplant.

Table S1. Characteristics of Younger Adult Kidney Transplant (KT) Stratified by Year of Transplant before and during the COVID-19 Pandemic

Characteristics	All Younger KT Recipients N=378,800	1990-1994 N=42,092	2010-2014 N=57,106	2015-3/14/2020 N=71,224	3/15/2020-2022 N=45,135	*P* Value
**Recipient factors**						
Age, mean ± SD	46.9±11.9	41.5±11.8	48.3±11.6	48.2±11.6	48.3±11.4	<0.001
Female, %	39.7	40.5	39.1	39.4	38.7	<0.001
Black, %	26.6	22.5	27.7	29	30.7	<0.001
Body mass index, kg/m^2, mean ± SD	27.9±5.7	24.9±5.0	28.3±5.6	28.3±5.6	28.7±5.7	<0.001
Hepatitis C virus positive, %	5.1	6.1	5.5	4.7	2.8	<0.001
Years on dialysis, median (IQR)	1.8 (0.4-4.1)	1.4 (0.5-2.7)	2.1 (0.3-4.5)	2.5 (0.4-5.4)	2.5 (0.4-5.2)	<0.001
Peak panel reactive antibody, median (IQR)	2.0 (0.0-13.0)	3.0 (0.0-12.0)	0.0 (0.0-23.0)	0.0 (0.0-24.0)	4.0 (0.0-48.0)	<0.001
Cause of end-stage renal disease, %						
Diabetes mellitus	24.5	21.0	25.5	26.2	28.1	<0.001
Hypertension	17.6	12.9	20.4	20.7	21.1	<0.001
Polycystic kidney disease	9.6	8.5	10.5	10.0	8.7	<0.001
Glomerulonephritis	25.5	32.3	24.3	23.9	22.8	<0.001
Other	22.8	25.3	19.3	19.2	19.3	<0.001
**KT factors**						
No human leukocyte antigen mismatches, %	9.1	11.3	6.9	4.6	4.2	<0.001
Kidney pumped, %[a]	31.1	13	40.1	46.3	50	<0.001
Cold ischemia time, median (IQR) [a]	13.0 (2.2-21.1)	18.0 (6.0-27.0)	10.5 (1.8-18.1)	11.8 (2.4-19.3)	15.7 (6.8-22.0)	<0.001
**Donor factors**						
Live donor KT, %	34.4	25.4	37.1	31.8	24.3	<0.001
Donor age, mean ± SD	37.7±14.8	33.8±15.6	38.7±14.6	38.6±14.5	38.8±14.0	<0.001
White race, %	70.0	77.9	67.4	67.5	66.7	<0.001
Donation after cardiac death, % [a]	8.6	0.3[b]	10.3	15.1	22.4	<0.001
Expanded criteria donor, %[a,c]	11.9	7.0	11.9	10.6	9.9	<0.001

SD = standard deviation; IQR = interquartile range.
The P-values compare the value of each recipient, donor, and transplant factor between 1990-1994, 2010-2014, 2015-3/14/2020, and 3/15/2020-2022.
[a]For deceased donors.
[b]For 1994-1996 first year donation after cardiac death was reliably recorded.
[c]Expanded criteria donors refer to older kidney donors (≥60 yr or 50-59 yr) and have two of the following three features: Hypertension, terminal serum creatinine >1.5 mg/dl, or death from cerebrovascular accident.

Table S2. Outcomes in Older Kidney Transplant (KT) Recipients by Sex

Year of KT		aHR (95% Confidence Interval)	
**Patient survival**	**N**	**Female**	**Male**
All donor (n = 73,078)[a]			
1990-1994	1,961	Reference	Reference
1995-1999	3,745	0.73 (0.54-1.00)	0.89 (0.71-1.10)
2000-2004	7,296	0.61 (0.45-0.83)	0.84 (0.68-1.04)
2005-2009	11,580	0.48 (0.36-0.66)	0.67 (0.54-0.83)
2010-2014	14,353	0.44 (0.32-0.60)	0.58 (0.46-0.72)
2015-3/14/2020	19,793	0.48 (0.34-0.68)	0.69 (0.54-0.88)
3/15/2020-2022	14,350	0.62 (0.43-0.89)	0.83 (0.64-1.07)
Deceased donor (n = 54,943)[a]			
1990-1994	1,749	Reference	Reference
1995-1999	2,963	0.69 (0.50-0.95)	0.87 (0.69-1.09)
2000-2004	5,018	0.53 (0.39-0.74)	0.84 (0.67-1.05)
2005-2009	8,410	0.45 (0.32-0.61)	0.66 (0.52-0.82)
2010-2014	10,651	0.40 (0.29-0.55)	0.54 (0.43-0.68)
2015-3/14/2020	14,712	0.45 (0.31-0.64)	0.67 (0.52-0.87)
3/15/2020-2022	11,440	0.57 (0.39-0.83)	0.81 (0.61-1.06)
**Death-censored graft survival**			
All donor (n = 73,078)[c]			
1990-1994	1,961	Reference	Reference
1995-1999	3,745	0.57 (0.39-0.85)	0.95 (0.70-1.29)
2000-2004	7,296	0.57 (0.39-0.83)	0.81 (0.60-1.10)
2005-2009	11,580	0.37 (0.25-0.55)	0.57 (0.42-0.77)
2010-2014	14,353	0.32 (0.22-0.47)	0.47 (0.35-0.64)
2015-3/14/2020	19,793	0.25 (0.16-0.39)	0.35 (0.24-0.50)
3/15/2020-2022	14,350	0.22 (0.13-0.36)	0.28 (0.19-0.41)
Deceased donor (n = 54,943)[c]			
1990-1994	1,749	Reference	Reference
1995-1999	2,963	0.54 (0.36-0.81)	0.91 (0.67-1.25)
2000-2004	5,018	0.55 (0.37-0.82)	0.80 (0.58-1.09)
2005-2009	8,410	0.35 (0.23-0.52)	0.54 (0.39-0.74)
2010-2014	10,651	0.32 (0.22-0.48)	0.45 (0.33-0.61)
2015-3/14/2020	14,712	0.24 (0.15-0.40)	0.35 (0.24-0.51)
3/15/2020-2022	11,440	0.22 (0.13-0.37)	0.29 (0.19-0.43)

Older recipients (age ≥65)
Cox models was assessed to estimate adjusted hazard ratios (aHRs) of mortality and death-censored graft loss over time relative to 1990-1994, while adjusting for recipient, donor, and transplant factors as listed below.

[a]Recipient factors (sex, age, race, body mass index (BMI), hepatitis C virus (HCV) status, preemptive KT, cause of end-stage kidney disease (ESKD), peak panel reactive antibody (PRA), number of years on dialysis), transplant factors (human leukocyte antigen (HLA) mismatches, cold ischemia time, kidney pumped, insurance type) and donor factors (race, age, hypertension, expanded criteria donor (ECD), donation after cardiac death (DCD), diabetes mellitus).

[c]Recipient factors (sex, age, race, BMI, HCV status, preemptive KT, cause of ESKD, peak PRA, number of years on dialysis), transplant factors (HLA mismatches, cold ischemia time, kidney pumped, and insurance type), donor factors (race, age, hypertension, ECD, DCD, diabetes mellitus, stroke as cause of death).

Figure S1. Younger adult kidney transplant (KT) recipients stratified by the year of transplant. The left y-axis (bar) shows the number of younger KT recipients, and the right y-axis (line) shows the percent of those aged <65 years out of the total KT recipients in that year.

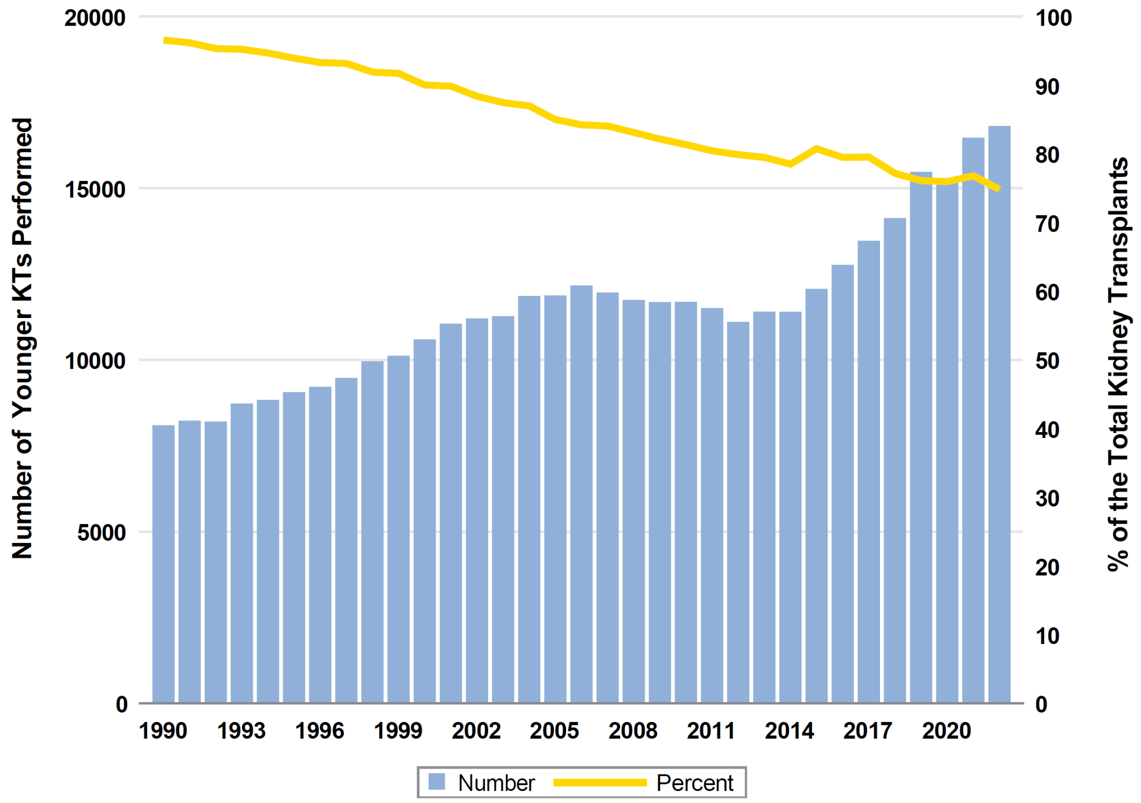

Figure S2. (A) Mortality in younger adult kidney transplant (KT) recipients, and (B) death-censored graft loss in younger KT recipients by the year of transplant. Transplants during the COVID-19 pandemic era denoted by the Black colored curve, which ends around 3 years due limited follow-up data in this cohort.

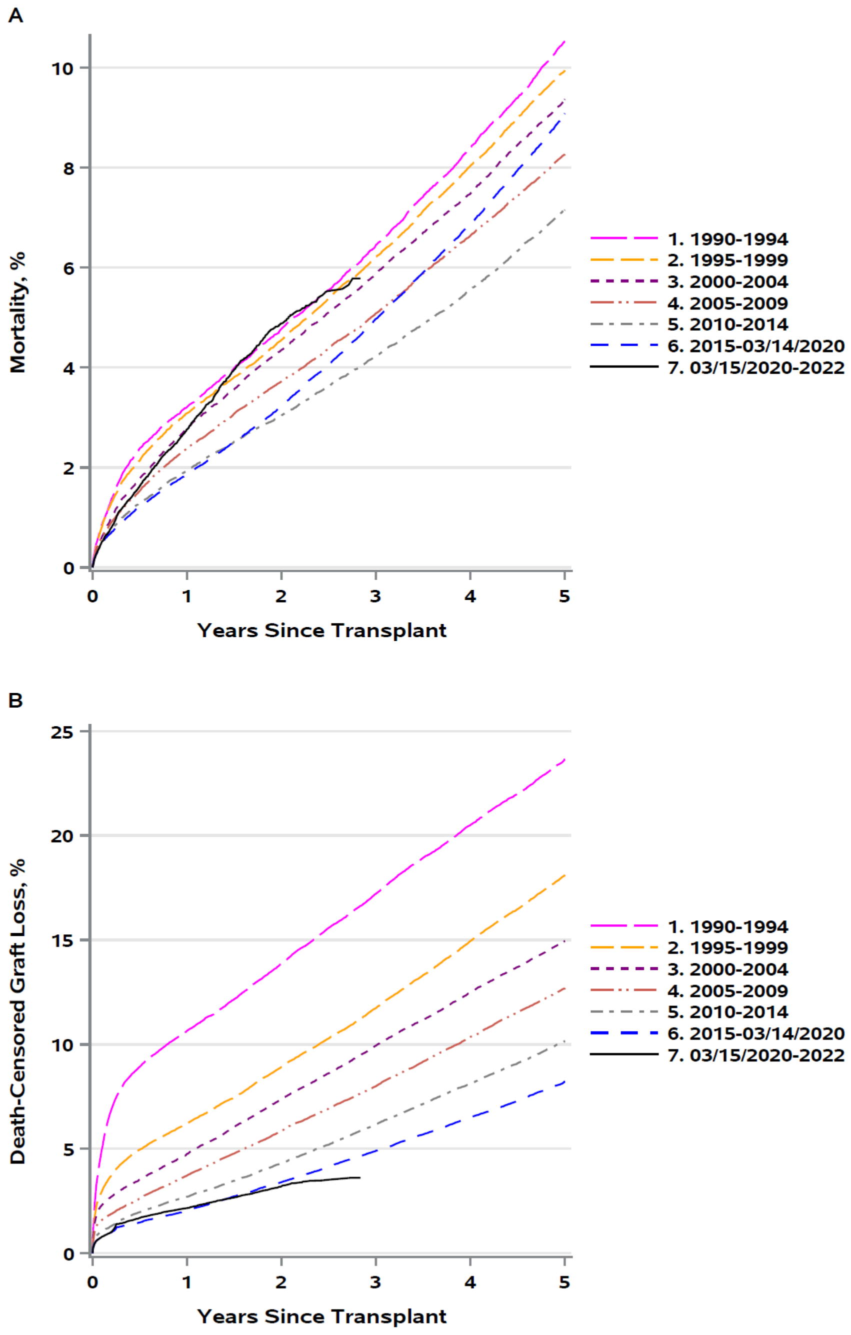

Supplement: Supplementary file 1 [file txd-9-e1520-s001.pdf]
